# Effect of facemask use on cognitive function during a maximal running aerobic fitness test

**DOI:** 10.3389/fphys.2022.912740

**Published:** 2022-09-29

**Authors:** Maamer Slimani, Armin Paravlic, Ensar Abazovic, Hela Znazen, Nicola Luigi Bragazzi

**Affiliations:** ^1^ Postgraduate School of Public Health, Department of Health Sciences (DISSAL), Genoa University, Genoa, Italy; ^2^ Higher Institute of Sport and Physical Education of Ksar Said, University of Manouba, Tunisia, North Africa; ^3^ Faculty of Sport, Institute of Kinesiology, University of Ljubljana, Ljubljana, Slovenia; ^4^ Science and Research Centre Koper, Institute for Kinesiology Research, Koper, Slovenia; ^5^ Faculty of Sports Studies, Masaryk University, Brno, Czechia; ^6^ Faculty of Sports and Physical Education, Sarajevo, Bosnia and Herzegovina; ^7^ Department of Physical Education and Sport, College of Education, Taif University, Taif, Saudi Arabia; ^8^ Laboratory for Industrial and Applied Mathematics (LIAM), Department of Mathematics and Statistics, York University, Toronto, ON, Canada

**Keywords:** COVID-19, facemask, exercice, neuropsychological Tests, coronavirus

## Abstract

**Background:** The aim of the present randomized, crossover study was to determine the physiological and cognitive function responses while wearing a facemask during a maximal running aerobic fitness test.

**Methods:** Fourteen healthy, nonsmoking physical education students (age = 17.5 years, height = 1.72 m, body mass = 70.4 kg) volunteered to participate in this study. They carried out a 20 m multistage fitness test (MSFT) while wearing or not a cloth facemask on two separate occasions performed in random order. The “Rating of Perceived Exertion” (RPE) and the d2 test for visual attention were administered and assessed before and immediately after the MSFT for both conditions (with or without a facemask).

**Results:** When wearing the facemask, the participants exhibited lower maximal aerobic speed (*p* = 0.039), VO_2max_ (*p* = 0.039), distance covered during the MSFT (*p* = 0.057), and concentration performance (*p* < 0.001), when compared with the control situation (without facemask). Moreover, they made more errors compared with the control condition (*p* = 0.021). The use of a cloth facemask during maximal endurance running tests (such as the MSFT) reduced VO_2max_, and measures of cognitive performance as assessed by the test of focused visual attention (the d2 test). This data suggests avoiding using a cloth facemask during maximal aerobic fitness tests, and before any tasks that require a high level of visual attention.

## 1 Introduction

Recently, the worldwide spread of the still ongoing “Coronavirus Disease 2019” (COVID-19) pandemic has profoundly disrupted the normality of daily life, forcing populations to practice social distancing and self-isolation. COVID-19 has directly impacted humans' health and affected their lifestyles, including physical activity, nutrition, and sleep behavior (Ammar et al., 2020; Znazen et al., 2021). The pandemic has had cascading and compound effects involving many domains and sectors of daily life, such as education, travel, and sport, globally, and resulted in canceling and/or postponing of many events, including major international sports events.

Given that the COVID-19 virus is primarily transmitted among people through respiratory droplets and contact routes, wearing a facemask was mandated in order to prevent the transmission and limit the spread of the virus, thus controlling and containing the infectious outbreak (Hopkins et al., 2021; Matuschek et al., 2020). At the same time, it has been reported that a prolonged use of facemasks may lead to physiological and psychological concerns (Li et al., 2005; Rosner, 2020; Epstein et al., 2021). Chronic use of facemasks may result in an increased hypoxia state, due to the decrease in blood oxygen saturation and increase in heart rate and transcutaneous CO_2_ (Li et al., 2005; Beder et al., 2008; Rebmann et al., 2013; Rosner, 2020; Scheid et al., 2020; Epstein et al., 2021). Moreover, it could consequently increase stress levels, mental fatigue, and reaction time, impairing responses to perceptual tasks (Modena et al., 2021; Grimm et al., 2022), resulting in headaches, and making communication more difficult and challenging (Rosner, 2020; Tornero-Aguilera and Clemente-Suárez, 2021; Lee et al., 2022).

Information about the effects of facemask on physiological and cognitive functions during exercise is limited (Shaw et al., 2021a; Slimani et al., 2021). It seems that wearing a mask during exercise imposes extra pressure on ventilation (Hopkins et al., 2021), which may lead to small changes in physiological parameters, such as increased dyspnea, end-tidal CO_2_, heart rate and respiratory rate (Shaw et al., 2021b; Hopkins et al., 2021). In addition, contradictory results have been reported on its effect on physical performance. A recent randomized controlled trial of healthy adults aged 18–29 years showed that the use of cloth facemasks led to a 14% reduction in exercise time and 29% decrease in VO_2max_, attributed to perceived discomfort associated with mask-wearing (Driver et al., 2022). The same authors demonstrated that, when compared with the no-mask condition, participants reported feeling increasingly short of breath and claustrophobic at higher exercise intensities while wearing a cloth facemask. In contrast, a systematic review of the literature and meta-analysis showed that wearing a facemask while exercising had no impact on exercise performance (Shaw et al., 2021b). This contradiction may be due to the type of mask worn and the exercise practiced (do Prado et al., 2022; Fikenzer et al., 2020). In addition, considering that cardiovascular fitness is strongly correlated to cognition (Hötting and Röder, 2013) as well as exercise and physical activity in general (Mandolesi et al., 2018), it is important to evaluate the impact of exercising while wearing a facemask on cognitive functioning. Therefore, the aim of the present study was to determine the physiological and cognitive function responses while wearing a facemask during maximal exercise.

## 2 Materials and methods

### 2.1 Participants

Fourteen healthy (9 males and 5 females), nonsmoking, physical education students (age = 17.5 years, height = 1.72 m, body mass = 70.4 kg) volunteered to participate in this study after being informed of the nature and of the possible risks associated with the experiment. We included only participants who 1) are active and healthy; 2) practice only physical education; 3) without co-morbidities like diabetes, hypertension, epilepsy, cardiac illness, asthma, and other respiratory illnesses; 4) have been inactive in the last 48 hhours before the physical activity session; and, 5) have accepted to be involved in the present study.

The cloth mask (Half and quarter masks, Tunisia) was a 3-layer comfortable elastic ear loop: extra-soft ear loops eliminate pressure on the ears with layers composed of non-woven fabric. It can be considered a facemask that is typically used by the general population (Matuschek et al., 2020). Due to its availability and usage frequency by the general population, this type of mask was used in this study. The ethical approval for this study was provided by local institutions and was conducted in accordance with the 1964 Declaration of Helsinki.

### 2.2 Protocols

The study was carried out by implementing a randomized, crossover design during the COVID-19 pandemic, when the government adopted a package of protective and behavioral measures, such as the closing of universities and schools; avoidance of physical contact, handshakes, hugs, and kisses; wearing of facemasks; and, practicing of social/physical distancing. Each participant completed three 20 m multistage fitness tests (MSFT): one for familiarization with MSFT, one with and one without wearing a facemask. The last two MSFTs were conducted in random order. Each test took place at approximately the same time of the day (±1.5 h) and was separated by at least 3 days. During the first test, anthropometric data were collected. Furthermore, participants familiarized themselves with cognitive tests. During all sessions, no drug was administered. All participants gave maximum effort and were encouraged to do so.

Additionally, the d2 test and the “Rating of Perceived Exertion” (RPE) test were administered at the two-time points (15 min before the MSFT and immediately after the test) across the exercise tests (with and without wearing a facemask) in order to assess cognitive function and perceived exertion. After a 10 min warm-up, participants performed the MSFT and responded to the RPE scale immediately afterward. Participants were advised to avoid exercise, and restrain from caffeine, and alcohol consumption 48 h before each laboratory visit. Moreover, throughout the study, participants were instructed to maintain their regular dietary habits and exercise regimens.

### 2.3 Evaluations

#### 2.3.1 The 20 m multistage fitness test

The MSFT was carried out as outlined in Léger et al. (1988) study. It was previously validated as a predictor of VO_2max_ (intra-class correlation coefficient = 0.90) in adult people (Chung et al., 2022). The participants ran backwards and forwards between two lines 20 m apart, in accordance with the recorded “beep” sound. Only completed shuttles were considered successful runs. The test commenced with an initial speed of 8 km/h, being gradually increased by 0.5 km/h every minute and was stopped if the participant did not reach the line (within 2 m) for two consecutive ends after being warned. Maximal aerobic speed (MAS) was computed as the velocity of the last successfully completed stage, associated with VO_2max_ for the shuttle run test. VO_2max_ was calculated using the Léger et al. (1988) equation.

#### 2.3.2 Attention assessment

The d2 test was used to determine the level of concentrated visual attention of participants (Brickenkamp and Oosterveld, 2012). It consists of 14 rows with 47 characters per line. These characters are the letters d or p, with a total of one to four dashes above and below each letter. Participants were asked to scan each line and cross out only the characters containing the letter d with two dashes during 20 s. After completion of the d2 test, two variables were calculated: concentration performance (CP) and the total number of errors made by the participants (E). CP is calculated as the number of correctly marked d2-symbols minus the number of incorrectly marked symbols (symbols that are not d2-symbols). The total number of E is assessed as the number of errors made by failing to correctly identify a d2-symbol plus the number of errors made by incorrectly marking symbols that are not d2-symbols. We considered both CP and E in the current study.

#### 2.3.3 Rating of perceived exertion

RPE scale was used to estimate the participants’ perceived effort. It ranged between 0 “no perceived effort” (i.e., rest) and 10, which corresponded to “maximal perceived effort” (i.e., the most stressful exercise ever performed) (Borg, 1998).

### 2.4 Statistical analysis

Descriptive statistical analysis was carried out by computing the means and standard deviations for each of the variables under study. The normality of data distribution was verified by applying the Shapiro-Wilk test, which was preferred over other normality tests, given the sample size employed in the present investigation. Differences for performance-related measures between conditions (wearing a mask or not) were assessed by classical Average-Based Change statistics (ABC) (Estrada et al., 2019), more specifically the Student's t-test for dependent (paired) samples. MainThe main effects for cognitive response (CP, and E), and RPE were studied with repeated measure general linear models (GLM) with condition (wearing a mask or not) and time (PRE and POST) as within factors. Moreover, post hoc analyses were carried out, correcting for multiple comparisons.

Depending on the standard deviations, Glass's delta or Hedge's g was calculated as effect size. The former was computed for performance-related measures. For cognition- and perceived exertion-related measures, the magnitude of the difference between values was measured by using Hedge's g. The computed effect size was interpreted as follows: trivial: 0.0–0.2; small: 0.2–0.5; moderate: 0.5–0.8 and large: ≥ 0.8 (Lovakov and Agadullina, 2021). All statistical analyses were conducted utilizing the commercial software “Statistical Package for Social Sciences” (SPSS version 27.0, IBM, Armonk, NY, USA). Results with *p*-values less than or equal to 0.05 were considered statistically significant.

## 3 Results

Regardless of condition they were allocated, significant alterations were observed for all measures of interest (all, *p* < 0.001) following MSFT.

### 3.1 Measures of physical performance

During the experimental condition, the participants showed a 7%, 11% and 17% lower MAS (Glass’s delta = −0.85; *p* = 0.039), VO_2max_ (Glass’s delta = −0.85; *p* = 0.039), and distance covered during the MSFT (Glass’s delta = −0.76; *p* = 0.057) when compared to the control condition, respectively (Table 1).

**TABLE 1:** Comparison of physical performance measures while wearing or not a facemask.

		With facemask (N = 14)		Without facemask (N = 14)		Glass’s delta	T value (*p* value)
Parameters		Mean	SD	Mean	SD		
Shuttle running (repetitions)		6.57	1.83	7.93	1.77	−0.76 (−1.55; 0.04)	−1.993 (0.057)
Maximal aerobic speed (km/h)		10.71	0.93	11.46	0.89	−0.85 (−1.64; -0.02)	−2.178 (0.039)
VO_2max_ (ml/min/kg)		36.89	5.61	41.39	5.32	−0.85 (−1.64; −0.02)	−2.178 (0.039)

### 3.2 Concentration performance

During both the experimental and the control conditions, the participants exhibited significantly lower CP following the MSFT, when compared with PRE (F_1,26_ = 393.558, *p* < 0.001, η^2^ = 0.938). There was a significant time × group interaction effect (F_1,26_ = 32.042, *p* < 0.001, η^2^ = 0.552). Post hoc analysis showed that, during the experimental condition, the participants experienced significantly greater decreases in CP compared with the control condition (−11% versus −5%, Hedges’ g = 2.08, *p* < 0.001) (Table 2).

**TABLE 2:** Comparison of cognitive function following the MSFT, conducted while wearing or not a facemask.

		With facemask (N = 14)		Without facemask (N = 14)		Hedges’ g (95% CI)	Main effect	Interactions
Parameters	Time	Mean	SD	Mean	SD		*p* value [η^2^]	*p* value [η^2^]
Concentration performance	PRE	163.29	11.83	164.64	11.88	2.08 (1.15; 2.98)	**<0.001 [0.938]**	**<0.001 [0.552]**
	POST	146.07	9.83	155.07	10.87			
Total number of errors	PRE	43.71	11.83	42.36	11.88	−0.90 (−1.65; −0.13)	**<0.001 [0.909]**	**0.021 [0.188]**
	POST	65.07	11.22	58.07	9.10			
Rating of Perceived Exertion	PRE	1.14	0.95	0.43	0.51	−0.54 (−1.27; 0.20)	**<0.001 [0.987]**	**0.001 [0.432]**
	POST	9.43	0.65	8.21	0.43			

η^2^—partial eta-squared; bold values—statistical significance.

### 3.3 Total number of errors

During both the experimental and the control conditions, the participants had significantly higher number of errors following the MSFT, when compared with PRE (F_1,26_ = 260.523, *p* < 0.001, η^2^ = 0.909). There was a significant time × condition interaction effect (F_1,26_ = 6.036, *p* = 0.021, η^2^ = 0.188). Post hoc analysis showed that, during the experimental condition, the participants made more errors compared with the control condition (49% versus 37%, Hedges’s g = −0.90, *p* = 0.021) (Table 2).

### 3.4 Rating of perceived exertion

During both the experimental and the control conditions, the participants had significantly higher RPE following the MSFT, when compared with PRE (F_1,26_ = 1931.651, *p* < 0.001, η^2^ = 0.987). There was a significant time × condition interaction effect (F_1,26_ = 9.520, *p* = 0.001, η^2^ = 0.432). Post hoc analysis showed that, during the experimental condition, the participants experienced a lower increase in RPE compared to the control condition, however, the observed difference was not significant (725% versus 1817%, Hedges’ g = −0.54, *p* = 0.155) (Table 2).

## 4 Discussion

The current study investigated the effects of wearing the facemask during maximal-intensity aerobic endurance testing on physical performance and cognitive function parameters. The main findings were that wearing a facemask negatively affected MAS, predicted VO_2max_, and cognitive function. Briefly, both concentration performance and the number of errors made decreased and increased to a greater extent during the experimental condition after the MSFT than during the control condition, respectively. Although the difference was not statistically significant, an interesting finding was that, during the experimental condition, the participants experienced group experienced a smaller increase in RPE after the MSFT than during the control condition group. This could be primarily due to the overall exercise volume because MAS and the distance covered during the MSFT were 7% and 17% lower during the experimental condition than during the control condition, respectively.

The results showed significantly lower MAS, VO_2__max_, and covered distance during the MSFT, while the participants wore the cloth facemask, thus indicating that wearing a mask reduces overall aerobic performance. We can assume that physical performance decrements were due to acute hypoxia, which can be explained by narrowing the arteriovenous oxygen difference and reduced maximal cardiac output (Peltonen et al., 2001; Lässing et al., 2020) but this claim needs further investigation. More likely, the primary reason for this could be attributed to perceived discomfort associated with mask-wearing. Namely, in a recent study by Driver et al. (2022), the participants reported feeling increasingly short of breath and claustrophobic at higher exercise intensities while wearing a cloth facemask. The ≈11% VO_2__max_ drop when wearing a mask compared to a mask-free environment emphasizes the concerns regarding facemask usage during high-intensity physical activity, but, still, these results are more mask-favorable than those by Driver et al. (2022), who observed a 29% decrease in VO_2__max_. For instance, the higher decrease in VO_2max_ in the study by Driver et al. (2022) compared with the present study may be due to the difference between exercise and environmental conditions (laboratory and field conditions). Furthermore, because the 20 m multistage fitness test was designed to determine the maximal aerobic power (Léger et al., 1988), these results further confirm the findings by Scott et al. (2018), who suggested that benefits from high-load exercise might not be augmented from additional hypoxia as for low- and moderate-load exercise.

There is strong evidence that regular physical activity and structured exercise interventions are associated with improvements in cognitive performance at both high and low intensity (Janssen et al., 2014). In the long-term view, these benefits are most likely related to improvements in cardiorespiratory fitness and consequently to cellular, molecular, and structural changes in brain regions involved in motor-cognitive function (Stillman et al., 2016). However, the benefits of short-term high-intensity exercise on cognitive function are inconclusive (Kao et al., 2017; Samuel et al., 2017), long with the mechanisms underlying those changes (Moreau and Chou, 2019). Our results complement previous findings (Alves et al., 2014; Samuel et al., 2017), showing the significant alteration in attention performance following maximal intensity exercise. Alves and colleagues (2014) showed that a single session of high-intensity interval training (HIIT) interval training reduced selective attention assessed using the Victoria Version of the Stroop test. We found that both groups experienced decrements in focused visual attention tasks whether or not the cloth mask was used, with the mask group showing significantly higher alterations than the no-mask group. The differences between the groups may be explained by various physiological factors, such as inhalation of exhaled CO_2_ that was mechanically trapped by the mask, which immediately triggers physiological adaptations to compensate for the lower O2 inhalation, similar to the condition during exercise in hypoxic environments (Peltonen et al., 2001; Lässing et al., 2020). In contrast, recent studies (Goh et al., 2019; Kim et al., 2016; Morris et al., 2020) showed that wearing a facemask during moderate, low and mild-intensity exercise had no effect on motor-cognitive performance. However, perceived dyspnea, manifested by 36% higher breathlessness, was observed when compared to the barefaced control group (Morris et al., 2020). In addition, another study investigated the effects of wearing a facemask during warm-up on attention (Slimani et al., 2021). Although positive changes in attention performance such as increased CP and decreased E were observed between wearing or not a cloth mask and no mask. Methodological differences between studies may have contributed to these discrepancies, as different types of facemasks (Modena et al., 2021) were used, eliciting lower or higher hypoxic environmental conditions. Also, different experimental conditions regarding the intensity of exercise, e.g., the maximum intensity of exercise until exhaustion was used in the present study versus moderate intensity of exercise involved in the study by Slimani et al. (2021) and/or prolonged sitting; and finally, different measures of interest were assessed in the studies by Samuel et al. (2017) and by Alves et al. (2014). These differences may account for some of the inconsistencies between studies that should be investigated in future experimental settings.

This study adds to the literature by providing new evidence about the effect of acute aerobic physical fitness tests with and without wearing a facemask on cognitive function. For example, a single bout of maximal aerobic exercise may have a negative effect on visual attention. Also, wearing a facemask during a physical fitness test can amplify this negative effect.

## 5 Conclusion

The use of a cloth facemask during maximal endurance running tests (such as the MSFT) reduced MAS, predicted VO_2max_, and distance covered, as well as measures of cognitive performance assessed by the test of focused visual attention d2. These results suggest avoiding cloth mask use in a sports setting, such as in an open skills, scenario (i.e., team sports), particularly in exercise with high intensity, and before any tasks that require a high level of visual attention. Further studies in different sports, evaluating the effects of prolonged, continuous, and high-intensity interval training while wearing a cloth facemask on physical performance and cognitive function are warranted.

## Data Availability

The raw data supporting the conclusion of this article will be made available by the authors, without undue reservation.
