# Erratum: Effect of facemask use on cognitive function during a maximal running aerobic fitness test

**DOI:** 10.3389/fphys.2022.1091789

**Published:** 2022-11-17

**Authors:** Frontiers Production Office

**Affiliations:** Frontiers Media SA, Lausanne, Switzerland

**Keywords:** COVID-19, facemask, exercise, neuropsychological tests, coronavirus

Due to a production error, **Affiliation 7** was displayed as “Department of Physical Education and Sport, College of Education, Taif University, Toronto, ON, Canada.” The correct affiliation is “Department of Physical Education and Sport, College of Education, Taif University, Taif, Saudi Arabia.”

The publisher apologizes for the mistake. The original article has been updated.

